# Challenges in Patient Discharge Planning in the Health System of Iran: A Qualitative Study

**DOI:** 10.5539/gjhs.v8n6p168

**Published:** 2015-10-26

**Authors:** Masumeh Gholizadeh, Bahram Delgoshaei, Hasan Abulghasem Gorji, Sogand Torani, Ali Janati

**Affiliations:** 1 School of Public Health, Tehran University of Medical Sciences, Tehran, Iran; 2 Department of Health Services Management, School of Management and Medical Informatics, Iran University of Medical Sciences, Tehran, Iran; 3 Department of Health Services Management, School of Management and Medical Informatics, Tabriz University of Medical Sciences, Tabriz, Iran

**Keywords:** effective hospital discharge planning, health system, Iran

## Abstract

**Background:**

One of the main factors relating to quality of hospitals is effective discharge planning. Discharge planning promotes the quality of inpatient care and reduces unplanned hospital readmission. The current study investigated the challenges of discharge planning observed in the health system of Iran.

**Methods:**

This qualitative research was conducted using a thematic and framework analyses to identify the challenges under each themes defined by the World Health Organization (WHO), to understand barriers in developing an effective discharge planning system in Iran health system. The data was collected from detailed semi-structured interviews and sessions of focus group discussions. This study involved 51 participants including health policy makers, hospital and health managers, faculty members, nurses, practitioners, community medicine specialists and other professionals of the Ministry of Health and Medical Education (MOHME). To reduce the bias and to increase the credibility of the study, evaluation criteria from Lincoln and Guba were used. All interviews and FGDs were recorded and transcribed, then analyzed by the software MAXQDA-11 and also manually.

**Results:**

According to the WHO health systems framework, challenges of effective hospital discharge planning were divided into six areas, leadership/governance, service delivery, information, financing, health workforce, and medical production(themes), in which there were 5,3,2,2,3,1 subthemes respectively.

**Conclusion:**

It is evident from the findings of this study that changes in the perspective of policy makers, health staff and managers, strengthening of systematic approach, and establishment of required infrastructures are essential for successful implementation of effective discharge planning in health systems in Iran.

## 1. Background

Discharge planning is a main concept for quality patient care and sustainability of health system. Often discharge planning is considered set of secondary activities to be completed at the end of a patient’s stay, therefore its quality can be compromised (LHIN, 2011). Discharge planning is a complicated process which is composed of assessment of patient during admission in the hospital, training the patient and their family, post-discharge follow-up and evaluation (Bull & Roberts, 2001). Studies have shown implementing discharge planning could lead to decreasing the length of stay (LOS) and readmission, lowering costs, ensuring the continuity of care in the community, improving patient’s mental health, patient satisfaction with members of carer team, improving disease outcomes and a safer patient transition to home (Shepperd et al., 2013). Discharge planning not only improves the quality of life in patients but also their families (Jackson, 1994). In addition to this, discharge planning promotes patient safety (Cook et al., 2000; Jack et al., 2009). Therefore, discharge planning is vital in a health system and lack of effective discharge planning is the main challenge in promoting the quality of care (RB & AJ, 2004). Gaps in discharge planning can lead to unnecessary readmissions which can be expensive and life threatening for patients (Tazhibi et al., 2011). In the US, the UK and Australian Heath system, effective discharge planning has been a priority for several decades. These countries have formulated policies and procedures of effective discharge planning simultaneously with a multidisciplinary approach and coordinating post-discharge care support to reduce the number of readmissions to the hospital (Birmingham, 2004). In recent years, rate of avoidable readmissions is an indicator of the quality of hospital care. Today, given the significance of this issue, decreasing the hospital readmissions rate is a major challenge for health systems’ managers (Hassan, 2001) and is a significant issue in Iran. In Iran, despite lack of precise readmissions rates, it is estimated to be around 20- 40% (Malek Afzali, 2005).

Lack of performance or improper performance in discharge planning and consecutively rising readmissions may be generated from a number of factors including inadequate assessment of patients ready to be discharged, incoherent discharge planning, failure in communication and information transfer between hospital and community-based physicians, inadequate post- discharge follow-up, or combination of some of the above processes (Minott, 2008). Recent studies have shown that there is no structured and effective discharge planning in the hospitals in Iran (Ghafari & Mohamadi, 2007). The studies a comprehensive system of effective discharge planning is necessary to reduce avoidable readmissions in hospitals (Tazhibi et al., 2011). The issue of discharge planning, follow-up and continuity of care have been considered by the Ministry of Health and Medical Education in Iran - Clinical Governance and Accreditation to promote the quality of care in Iran. It is essential for health mangers and policy makers to pay attention to barrier s of effective discharge planning, for successful implementation of discharge planning. Therefore the focus of the current study is to investigate the challenges in the discharge planning faced by the health system in Iran.

## 2. Methods

The current qualitative study was conducted using a thematic and framework analyses to obtain the collective view of professional of health system in Iran. The study population consists of health policy makers, hospital and health managers, faculty members, nurses, community medicine specialists and other professionals of the Ministry of Health and Medical Education (MOHME).

In this study, purpose-based and snowball sampling method was utilized to assemble the collective insights of individuals involved in the study. The data was collected from semi-structured interviews. A guide was given to interviewees including nine general questions and a summary clarifying purpose of the interview. The data was collected from 51 participants, from semi-structured interviews and focus group discussion (FGD). First 25 participants participated in four FGDs, and 26 members participated in of which 14 did face-to-face interview and other 12 did semi-structured interviews.

The time and length of interview was set accordingly and the participants were assured of confidentiality and allowed to withdraw from the study at any time inspite of their given written consent at the beginning of the study. All interviews were conducted in quite places allowing for accuracy, precision and privacy purposes.

The length of interviews in FGDs and semi-structured interviews was approximately 90-120 and 30-60 minutes, respectively. All interviews were recorded using two electronic devices permission was obtained from all participants prior to recording. All recordings were listened twice and annotated as written documents by researchers. Primary substantive codes were extracted from the recordings and similar codes were categorized together. In addition to the recordings key statements, nonverbal and facial gestures were considered when transcribing recorded files.

Data analysis was completed both manually and using a software MAXQDA-11. Data was analyzed to identify barriers in implementing effective discharge planning in Iran health system. With the intention of raising the credibility Lincoln and Guba’s method of evaluative criteria were exerted including prolonged engagement, combination of data collecting method like interview, field note, reviewing the transcriptions and agreeing on codes and classes extracted by researchers and peer reviewed, typed notes to remove any ambiguity of interviewees.

The interviews, determination of codes and classification was done by several officials experience in qualitative research, who do not have any conflict of interest with this study.

## 3. Results

Individual interviews and focus group discussions were analyzed by with experienced experts and faculty members involved with hospital discharge planning. The analysis led to presentation principal themes, subthemes and items.

### 3.1 Leadership/Governance

In the discharge planning system, leadership/governance is one of the most important functions and components of health system. Leadership or stewardship means determination and reinforcement of executive regulations, policymaking and formulating of strategies for all stakeholders in discharge planning system, acceptance of responsibility and accountability in the highest level (MOHEM 2010). Findings in Table 1 consist of five subthemes that are explained briefly under the leadership theme.

**Table 1:** Challenges of effective hospital discharge planning in the area of leadership/governance

Themes	Subtheme	Item
LEADERSHIP/Governance	Systematic Approach	Giving no priority to the discharge planning in heath system Lack of comprehensive plan to develop discharge planning in the ministry of health Lack of patient-centered approach in the health system Lack of systematic and comprehensive approach to the plan Disregarding the holistic care in training curriculum

	Structure	Lack of referral system and rationing of health services system Lack of structured, systematic and coordinated system Lack of comprehensive benefit package Lack of specific standards, rules, regulations on discharge planning

	Management	Low willpower of senior managers Poor monitoring and evaluation system Poor and inefficient management in heath system Managers and policy makers’ inaccurate comprehension of discharge planning

	Communication	Poor inter and intra sectional contribution Lack of defined interaction and communication among medical personnel to provide care Weakness in written supportive policies on discharge planning

	Advocacy	Lack of home care system in health system Lack of powerful supportive centers (such as community-based organizations)

Identified subthemes in this of the area include systematic approach, structure, management, communication, and advocacy are explained in detail below.

#### 3.1.1 Systematic Approach

In Iran health system there is lack of comprehensive and systematic approach to patient care which has led to scattered and fragmented discharge planning. Discharge planning in hospitals in Iran is an isolated event, instead of a process. Therefore, there is no interaction among providers of services, which creates difficulties in implementation of discharge planning. Accordingly, participants in interviews stated that “*…As long as discharge planning is separated and divided duties* instead of *a process*, *it is difficult to develop an efficient and effective discharge planning* “(p. 44). Most participants expressed that discharge planning has not been a priority area in the health system of Iran. Therefore has not been in the strategic plan of the health system. One of the participant expressed that: “*One of the main barriers, in my opinion, is the attitude of health system. Discharge planning has not been a priority area in health system of Iran because it has treatment-based attitude rather than health promotion*” (p. 25). Moreover, according to the views of the majority of participants, discharge planning is not included in training curriculum of medical and paramedical students.

#### 3.1.2 Structure

Unsuccessful implementation of referral system and no defined administrator for discharge planning in different levels of health services have caused ambiguity and confusion for patients. In such a system, there would not be possibility of improvement in discharge planning. Therefore, most of interviewees declared that: *“…firstly, a referral system is required for discharge planning. There are Obstacles in building a referral system*”. (p. 33). Appropriate planning and organizing is essential for successful implementation of any program. Most participants were unanimous in that there is no integrated and structured system of discharge planning in Iran. One of the participant mentioned that: *“…discharge planning does not have a coherent structure in health system of Iran. This is being carried out on personal desire instead of a legally mandated function …*” (p. 8). To establish an effective discharge planning policy, guidelines of this plan and service package of discharge planning are also as stated by the participants of the current study.

#### 3.1.3 Management

Low levels of determination and commitment in senior managers, lack of empowerment in managers to implement effective discharge planning, low familiarity and attitude of senior managers about discharge planning were the most significant subthemes emphasized by participants. “*Discharge planning has no priority due to low familiarity among senior managers*.” (p. 46). “*In health system, attitude of managers and policy makers plays an essential role. According to the managers the hospital has no responsibility for post discharge care.” (p. 22). “Discharge planning has been announced as a standard for accreditation which now needs to be implemented. However, empowerment of managers is essential for successful implementation of effective discharge planning*…” (p. 32). Other main barriers of discharge planning mentioned by majority of the participants was lack of monitoring and evaluation system of discharge planning due to indefinite and unclear plan of discharge planning in health system of Iran.

#### 3.1.4 Communication

Inter sectional and Cross sectional communication is one of the main factors in successful implementation of discharge planning. Participants declared that *“… other organizations like private organizations, nongovernmental organizations and charities should contribute in implementation of this plan*.” (p. 49). “…*staff at the first level of providing services, primary health care (PHC), can help hospitals to follow up patients actively*.” (p. 46). Lack of defined communication for transition of care among various levels of care providers and among team members of discharge planning in hospitals as mentioned by majority of the participants. Another item expressed by participants is deficiency in written supportive policies supporting this implementation strategy.

#### 3.1.5 Advocacy

Establishment, reinforcement and utilization of supportive centers such as nongovernmental organizations (NGO) and private organizations such as home care agencies will be valuable in stabilizing the plan. A large number of respondents agreed on the necessity of these supportive centers in discharge planning “*Home care and family nursing facilities are essential to support this plan*.” (p. 29). Additionally, majority of participants expressed that lack of support by senior managers and policy makers of discharge planning is one of the key barriers of implementation of this plan.

### 3.2 Service Delivery

The main goal of health systems is health promotion among patients and their families. The fundamental function of health systems to meet this goal is delivery of health service.

Results obtained from content analysis of interviews in the second area have been listed in Table 2. As shown by the results, there are three subthemes (structure, attitude, and standard) explained in further detailed below.

**Table 2:** Challenges in effective hospital discharge planning in the area of service delivery

Themes	Subtheme	Item
SERVICE DELIVERY	Structure	Poor communication among healthcare providers Insufficient physical space in hospital Poor system of patient training Lack of follow-up system Overloaded number of patients in hospital lack of comprehensive service delivery
	Attitude	Cultural differences related to home visit and discharge planning Lack of systematic approach to health delivery Lack of belief in continuing the care and completing the treatment Patients’ low trust in medical personnel No need for discharge planning in patients’ opinion
	Standard	Lack of clear ethical considerations on post-discharge follow-up Lack of guidelines on service delivery

#### 3.2.1 Structure

Pproper organization is essential for effective service delivery. In order to implement effective discharge planning well developed structure of service delivery is critical. Majority of participants were concerned about poor patient training at the time of discharge. “*In our hospitals, what we do about training and follow-up is simply provide a pamphlet for patients and ask them to return to the clinic by two weeks*. (p. 3)”. “*We don’t have patient follow up system. As the patient leaves the hospital, we have nothing to do with them*.” (p. 46). Most of the participants stated that there is inadequate physical space in hospitals and hospitals are overloaded with patients, and these are the major barriers in implementation of discharge planning. Overall as commentated by the participatants, “*lack of follow-up clinics in hospitals’is a structural barrier in the implemention of the plan*.” (p. 43).

#### 3.2.2 Attitude

Change in the attitude of all stakeholders (including patients, providers and administrators) is essential for the success of discharge planning. “*Characteristics of patients and the degree of importance of their health and their willingness to pay can be an obstacle to carry out the program…*” (p. 31). “… Distrust of patients on medical personnel may be one of main barriers. “*When we tell the patients, that we want to follow up, they think that we are solely doing it for money*” (p. 25). According to majority of participants, hospital managers are another important barrier. “*Managers have accepted the role of treatment lies just in the hospitals and continuity of care is not the responsibility of the hospital* (p. 42)’.

#### 3.2.3 Standard

It is necessary to develop standards and procedures in order to succeed in implementation of discharge planning. Lack of clear policies and guidelines relating to patient care during discharge planning leads to execution of programs on personal desire. One of the participant stated: “*Another problem is related to our guidelines. There are various and personalized methods followed by physicians that even nurses are not aware of and hence are unable to educate/train the patient*. “(p. 21). Additionally, absence of a clear definition of ethical considerations regarding post-discharge care and patient follow-up was extracted as another subtheme from interviewees. *“…Identified ethical and cultural consideration of discharge planning is required for successful implementation of discharge planning” (p. 31)*.

### 3.3 Information

Information is another component of health care organizations framework, which plays a crucial role in effective discharge planning. Results in Table 3 represent two sub themes and six subthemes in relation to the third area that is explained in details below.

**Table 3:** Challenges of effective hospital discharge planning in the area of information

Themes	Subtheme	Item
INFORMATION	Facilities	Poor and inappropriate hospital information system Lack of integrated hospital information system (HIS) Lack of effective communication between hospital care and community care Lack of electronic health file

	Training	Lack of up-dated training materials Community and patients’ unfamiliarity with discharge planning

Two sub themes, named facilities and training, were discovered as discharge planning challenges in this area.

#### 3.3.1 Facilities

Implementation of any program is impossible without providing necessary facilities. Majority of the participants affirm that: “*electronic health records and proper implementation of family physicians are infrastructures for discharge planning, in Iran health system we have none*.” (p. 43). “*Each hospital has its own health information system (HIS) and we can’t really link them. In fact, we don’t have coherent and integrated health Information* (p. 17).

#### 3.3.2 Training

The most important part of discharge planning is training. In Iran health systems lack of appropriate training materials along with scientific developments can be assigned as one of barriers to the plan. Some participants commented: “*Educational content must be in accordance with scientific developments and must be revised on regular basis; however, unfortunately it is not practiced in Iran* (p. 24)”. Inadequate training and education of patients and families in relation to the importance of discharge planning was believed to be a hindrance in implementation of the discharge planning.

### 3.4 Financing

Managing financial resources is another vital component of medical and health organizations framework. In order to provide efficient health care requires a balanced and equitable distribution of financial resources to deliver health services (16).

Results in Table 4 demonstrate two sub themes and six items which are described in further detail.

**Table 4:** Challenges of effective hospital discharge planning in the area of financing

Themes	Subtheme	Item
FINANCING	Allocation	Insufficient budget and financial resources Poor insurance support for the plan Lack of efficient insurance systems

	Payment	High out-of-pocket payment (OPP) Lack of tariff for discharge planning services Unfair payment system between staff and physicians (fee for service)

#### 3.4.1 Allocation

Most of the participants stated that shortage of financial resources is an extremely enormous obstacle which is considered as one of the critical barriers of discharge planning in Iran health system. Significant financial resources of health system belong to the health insurances whose payment policies play a critical role in the service delivery. Majority of the participants stated that health insurance support in necessary to prevent inequality in access to health services. Therefore, a participant states that *“In our country, health insurance does not accomplish its role and hence do not function very well…*” (p. 18). As asserted by a participant, another obstacle in implementing this plan is lack of national health insurance. One of the participants mentioned that “*most often, even if a hospital is willing to do discharge planning and follow-up, the patient has no intention, because the out of pocket expenses are huge*.”(p. 33).

#### 3.4.2 Payment

Health system has to design a suitable payment system to control health expenses and motivate providers. As stated by a respondent, “*the other obstacle is the payment system, where fees arecharged for services. This needs to be altered into other proper systems…*”(p. 43). “The service tariff is absolutely limited to prescription medicines, surgery and other in-the-hospital services.” (p. 46). On the other hand, according to some participants, inequitable distribution of financial resources among service providers is a main challenge. One respondent said, “*… The budget is distributed unequally wherephysicians are allotted 90 percent of the budget. However, others do their utmost in carrying out the program and are still under paid*.” (p. 25).

### 3.5 Health Workforce

The results in Table 5 depict three sub themes and eight items, related to the fifth area of WHO’s system building blocks. Full details are revealed below.

**Table 5:** Challenges of effective hospital discharge planning in the area of health workforce

Themes	Subtheme	Item
HEALTH WORKFORCE	Empowerment	Lack of qualified Health workers to initiate discharge planning Staff and managers’ poor knowledge of the program

	Motivation	Staff and physicians’ resistance to change on the implementation Physicians’ poor contribution on the implementation Staff and physicians’ inadequate motivation Lack of the spirit of team work

	Management	Discrepancy between workforce and workload Vagueness of workforces’ roles and duties

In this regard, challenges of discharge planning are composed of empowerment, motivation and management that are explained below.

#### 3.5.1 Empowerment

Human resources knowledge and capabilities are vital in implementation of discharge planning. Large number of participants believed that having sufficient and capable personnel is necessary to implement and stabilize the discharge planning system. One of the participants stated that “*… It is imperative to train technical and well-qualified workforce to implement dischargthe planning* “(p. 27). Currently there is poor knowledge of the program among staff members and managers as agreed by majority of participants in the study.

#### 3.5.2 Motivation

Motivated staff is always required to run a health program including discharge planning. Staff participants of the study informed that: “*If we perform the discharge planning for the following day, and we don’t have its infrastructures, then we may not have the incentive and related skills to execute it*” (p. 25). Additionally, “*in such implementations, lack of physician’s cooperation can also become a barrier. Discharge planning is a team work, and we alone won’t be able meet to the target if the physicians are not involved*” (p. 6).

#### 3.5.3 Management

Most of the interviewees agreed on the necessity of consistency between workforce and workload. “*…We have a little workforce and a lot of workload. In order to raise the quality of work, which is important for successful implementation of discharge planning, we need to meet standard man power requirements (p. 25). Furthermore*, some of the participants pointed out the confusion of what duties are performed by staff members. “*If we implement effective discharge planning, without defining duties for staff members, there will be difficulties in evaluation of the plan*.” (p. 11)

### 3.6 Medical Production

The results obtained from the content analysis of interviews in the sixth area on the basis of WHO’s system building blocks are prepared in Table 6.

**Table 6:** Challenges of effective hospital discharge planning in the area of medical production

Themes	Subtheme	Item
MEDICAL PRODUCTION	infrastructure	Lack of communication and electronic follow-up system Lack of technology to implement discharge planning Insufficient medical equipment to implement discharge planning

#### 3.6.1 Infrastructure

This area of discharge planning challenges presents one sub theme, called infrastructure. A majority of the respondents, indicated lack of communication and electronic system which is necessary to track patient records. “*We have to keep in touch with our patient via telephone, mobile phone, and internet and so on after being discharged from the hospital. The communication way should be specified and informed to the patients well in advance*.” (p. 42). “*The chief obstruction in this subtheme islack of essential software and network to record case information*.” (p. 27). The necessity of adequate equipment including wheelchair, walker, blood pressure monitoring device, glucose meter and other facilities required in discharge planning were also emphasized by the participants.

## 4. Discussion

The aim of the current study was to recognize challenges involved in discharge planning to improve the quality of care in Iran health system. In the area of leadership or governance, a systematic approach (top of the organizational hierarchy to the bottom) for senior managers or operational staff in healthcare system of Iran is crucial in policy planning and providing concrete services for discharge planning and to maximize organizational effectiveness (Wong et al., 2011). The other main challenge in this theme was the need to develop an effective and comprehensive discharge planning in healthcare system in Iran. Discharge process facilitated with a structured, systematic and coordinated system ensures a smooth patient transition from hospital to the community and improved patient health outcomes (Yam et al., 2012). Standardized discharge planning policy driven guidelines are being launched in the UK, US, and Australia, for healthcare staff to execute the process (Wong et al., 2011). One of the extensive challenges outlined in the present study is thelack of prioritization of discharge planning in the strategic plans of health care system in Iran. In conjunction with this study, other researches demonstrated that inappropriate assessment of priorities and allocation of financial resources in the ministry of health (Rajabi, 2011). Another main discharge barrier was lack of social support systems such as home care follow upto support of discharge planning. Lack of formal relationship between levels of care (referral system) was a major problem in the implementation of the discharge planning as stated in other studies (Coleman, 2003; Leathard, 2004; Kripalani et al., 2007; Birmingham, 2004). In the area of leadership of discharge planning, another challenge was the necessity to develop monitoring and evaluation system for discharge planning (Evans & Hendricks, 1993; Solomon et al., 2012). Inter-sectoral and cross-sectoral coordination in the health sector are also challenges faced in the discharge planning. Studies show that Cross- sectoral collaboration and communication are crucial in sharing of health information and thereby promote coordination of post-discharge care (DiGiacomo et al., 2010). In addition, Inter-sectoral collaboration also required for effective discharge planning (Weinberger et al., 1996; Glendinning & Rummery, 2003).

The main challenge of service delivery is the lack of standardized guidelines and package of discharge planning services which is also supported by previous studies to minimize discharge failures (Ghafari & Mohamadi, 2007; Greenwald et al., 2007). The main challenge of the health workforce theme is the lackof multidisciplinary team for effective implementation of discharge planning. Other study also showed that for implementation of effective discharge planning, good team work and leadership are absolute essentials (Cook et al., 2000; Pethybridge 2004). The major challenge identified and emphasized during the study is the shortage of employee’s familiarity with the discharge planning and lack of in-service training (Watts et al., 2006; Ghafari & Mohamadi, 2007). Majority of the existing staff members are resistant to changes in relation to discharge planning in the work force and this finding was also supported by previous studies (Hultberg et al., 2005). One of the key challenges in this area of current study was absence of for clear role and responsibilities for each staff member in the workforce and care coordination. A clear role of each member of the health care team (with a clear job description) in relation to discharge planning is not only beneficial for patients but also for the health system (Wong et al., 2011). Hospital discharge planning is a complex process that requires unified communications among hospital’s carer team, primary care teams, social services, patient care and the patient himself (Coleman, 2003; Cindy & Rockville, 2011). Coordination is essential for implementation of discharge planning, at all levels including among multiple providers, providers and patients and their families only is care coordination needed (Coleman, 2003; Bodenheimer, 2008). In the theme of information challenges include lack of proper information systems in hospital and maintenance of all health records electronically. Further studies prove that softwares used in medical centers are far from their real functions in hospital information management systems (HIS). Current HIS is not applicable for strategic decision support, control and improvement of hospital performance (Coleman, 2003; Rozbehani et al., 2012). Data from other studies suggests that one of the principal infrastructures of implementation is the integrated HIS and electronic health records, vital in improving the quality of patient information transfer (Thraen et al., 2012; Tyler et al., 2014). Moreover, another it is essential to improve training materials to ensure better self-care after being discharged from hospital. Results of similar studies are indicative that there is an increase in self-care responsibility for patients and their families as they return home (Kripalani et al., 2007). The main challenge being faced in the financing theme is the need to develope financial resources for successful implementation of discharge planning. In accordance with additional studies, financial obstacles can impose constraints on post-discharge care in hospitals. However, other studies showed that although higher costs are associated with improved quality of plans, the discharge planning not only lowers expenses but also improves the quality of healthcare for patients (Peikes et al., 2009). This study argues that today, effective discharge planning is an investment for hospitals, as it may lead to reduction of readmissions and complication rates, therefore leading to decrease in the future costs on the health system (A.K, Orav et al. 2009; Alper, O’Malley et al. 2014). In the area of payment, there is lack of support from health insurance companies was a main challenge that was investigated in this study in harmony with other studies (Mamon, Steinwachs et al., 1992; Coleman, 2003). The need to determining tariffs for discharge planning services was an addition challenge in the implementation of this plan (Naylor et al., 1994; Coleman, 2003).

In the medical production theme the main challenges were absence of communication and electronic follow-up technologies (telehealth or Telemedicine system; and telemonitoring technologies) and medical equipment to implement discharge planning. Implementation of effective discharge planning requires some infrastructures such as electronic follow-up requirements such as email or telephone, equipment required for post discharge care including walker, wheelchair, eyeglasses, hearing aids, security alarm, blood pressure monitoring device, glucose meter etc however, further studies are required (Dudas et al., 2001; (MOHLTC) 2011, Yam, Wong et al. 2012).

## 5. Conclusion

It is evident from the current study that implementation of discharge planning system in Iran encounters a variety of challenges. These challenges require serious attention by managers and policy makers in the system to turn these obstacles into opportunities for the health system nationwide. Furthermore, strategies to create and promote discharge planning needs to be adopted along with the aid of various policies, programs, supportive legislation. All of the above can work effectively in the advancement of the society’s health, improvement of patients, clinical outcomes and reduction in the costs of effective health system.
